# Retracted: Mannosylated Chitosan Nanoparticles for Delivery of Antisense Oligonucleotides for Macrophage Targeting

**DOI:** 10.1155/2021/1878130

**Published:** 2021-02-01

**Authors:** BioMed Research International

*BioMed Research International* has retracted the article titled “Mannosylated Chitosan Nanoparticles for Delivery of Antisense Oligonucleotides for Macrophage Targeting” [1] due to signs of image manipulation.

As noted on PubPeer [2], Figure 15(a) and (b) show signs of repeated duplication of figure elements. The authors provided additional data for other results reported in the article, though not the underlying measurements, and said the similarity of nanoparticles in Figure 15(b) was due to the “homogeneous nature of nanoparticle formulation in given size range.” The authors did not respond to retraction.
