# Supplementary material for: Retracted: Mannosylated Chitosan Nanoparticles for Delivery of Antisense Oligonucleotides for Macrophage Targeting
Source: Biomed Res Int. 2021 Feb 1;2021:1878130. doi: 10.1155/2021/1878130 (PMC7910040; doi:10.1155/2021/1878130)
Supplement: Supplementary Materials — Additional data for figures and tables. [file 1878130.f1.docx]

**Raw Data for figure and tables for manuscript**

Gyati Shilakari Asthana, Abhay Asthana, Dharm Veer Kohli, and Suresh Prasad Vyas, titled “Mannosylated Chitosan Nanoparticles for Delivery of Antisense Oligonucleotides for Macrophage Targeting, submitted in BioMed research international. Volume 2014, Article ID 526391, 17 pages,

**Fig 7: Stability of MCHODN NP2 formulations in 10 mM phosphate buffer containing 150 mM NaCl**

**Tabular data of fig 7 Stability of MCHODN NP2 formulations in 10 mM phosphate buffer containing 150 mM NaCl**

| **Time (day)** | **Turbidity (absorbance at 340 nm)** |
| --- | --- |
|  | **MCHODN NP2** |
| 0.25 | 0.351 |
| 0.5 | 0.350 |
| 1.0 | 0.348 |
| 1.5 | 0.347 |
| 2.0 | 0.339 |

**Fig.9: Cell Viability studies of MCH at different polymer concentration in Raw 264.7 and Hela cells (n=3)**

**Tabular data of Fig.9: Cell Viability studies of MCH at different polymer concentration in Raw 264.7 and Hela cells (n=3)**

| Polymer concentration[μg/ml] | Cell Viability (of % Control) of MCHNP | |
| --- | --- | --- |
|  | Hela cells | Raw264.7 cells |
| 0[untreated cell] | 100 | 100 |
| 5 | 106.4±1.2 | 99.2±1.8 |
| 10 | 100.2±2.2 | 104.5±2.4 |
| 20 | 91.6±1.4 | 97.2±2.8 |
| 30 | 74.8±1.8 | 86.8±3.2 |
| 50 | 57.6±1.6 | 72.4±2.4 |
| 100 | 63.2±1.4 | 85.0±3.2 |
| 150 | 61.8±1.0 | 86.2±1.4 |
| 200 | 60.2±1.4 | 88.4±1.2 |

**Fig. 10: Cell viability study of MCHODN NPs with different ODN concentration in Raw 264.7 cells after 24 h (n=3)**

**Tabular data of Fig. 10: Cell viability study of MCHODN NPs with different ODN concentration in Raw 264.7 cells after 24 h (n=3)**

| **ODN content (μg)** | **Cell Viability (of %Control)** |
| --- | --- |
| 0 | 100 |
| 1 | 92.5±7.1 |
| 5 | 85.4±10.6 |
| 10 | 78.6±10.4 |

**Fig 11: Transfection efficiency of MCHODN Nanoparticles at different N/P ratio in Raw 264.7 cells and Hela Cells (n=3)**

**Tabular Data of Fig 11: Transfection efficiency of MCHODN Nanoparticles at different N/P ratio in Raw 264.7 cells and Hela Cells (n=3)**

| **N/P ratio** | **ODN (ng/10^6^ cells) Raw 264.7 cells** | **ODN (ng/10^6^cells) Hela cells** |
| --- | --- | --- |
| Naked ODN | 24.12± 1.4 | 26.32± 1.2 |
| 1 | 28.78± 1.2 | 25.58 ± 1.2 |
| 3 | 73.18± 4.2 | 28.86±1.4 |
| 5 | 63.08± 3.6 | 26.28±1.6 |
| 7 | 59.12*±2.4 | 23.14±0.81 |

**Fig. 12: Comparative study of Transfection efficiency in Raw 264.7 cells and Hela cells with free ODN and MCHODN NP2 and at N/P ratio of 3:1 (n=3). Transfection efficiency using Lipofectin 2000 was set as a positive control.**

**Tabular data of Fig. 12: Comparative study of Transfection efficiency in Raw 264.7 cells and Hela cells with free ODN and MCHODN NP2 and at N/P ratio of 3:1 (n=3). Transfection efficiency using Lipofectin 2000 was set as a positive control.**

| **Formulations** | **ODN (ng/10^6^ cells) Raw 264.7 cells** | **ODN (ng/10^6^cells) Hela cells** |
| --- | --- | --- |
| Free ODN | 24.12± 1.4 | 26.32± 1.2 |
| MCHODN NP2 | 73.18± 4.2 | 28.86±1.4 |
| Lipofectin/ODN | 76.84±4.5 | 92.84±4.5 |

**Fig.13 Competition assay of MCHODN NP2 (N/P=3) in Raw 264.7 cells with different concentration of mannose.**

**Tabular data of Fig.13 Competition assay of MCHODN NP2 (N/P=3) in Raw 264.7 cells with different concentration of mannose.**

| **Mannose concentration (mM)** | **ODN(ng/10^6^ cells)** |  |
| --- | --- | --- |
|  | MCHODN NPs | |
| 0 | 70.18 ± 1.2 | |
| 10 | 39.18±2.6 | |
| 20 | 35.24±2.4 | |
| 50 | 20.12±1.8 | |
| naked ODN | 24.12±1.6 | |
| Lipofectin/ODN | 73.84±4.2 | |

**Fig. 14: TNF-α expression profile of transfected Raw 264.7 cells with free ODN and**

**MCHODN NP2 after stimulation of cells with 100 ng/ml LPS (n=3),**

**^#^ *Control cells treated with PBS*+*LPS***

**Tabular data of figure 14. TNF-α expression profile of transfected Raw 264.7 cells with free ODN, CHODN NP2 and MCHODN NP2 after treatment of cells with 100 ng/ml LPS (n=3)**

| **Cell treatment** | **TNF-α content (% of ^#^control)** |
| --- | --- |
| Free ODN+LPS | 92.42±2.4 |
| CHODN NPs+ LPS | 72.36±2.2 |
| MCHODN NPs+ LPS | 43.96±2.6 |
| MCHODN RD NPs+ LPS | 108.12±4.5 |

^#^ *Control cells treated with PBS*+*LPS*
